# Duplication of the Posterior Cerebral Artery (PCA) or “True Fetal PCA”: An Extremely Rare Variant

**DOI:** 10.5334/jbsr.1502

**Published:** 2018-02-20

**Authors:** Bruno Coulier

**Affiliations:** 1 Clinique Saint-Luc, Bouge, BE

**Keywords:** Posterior cerebral artery, Fetal variant, 3D angio-CT, Circle of Willis

## Case

**Figure 1:**
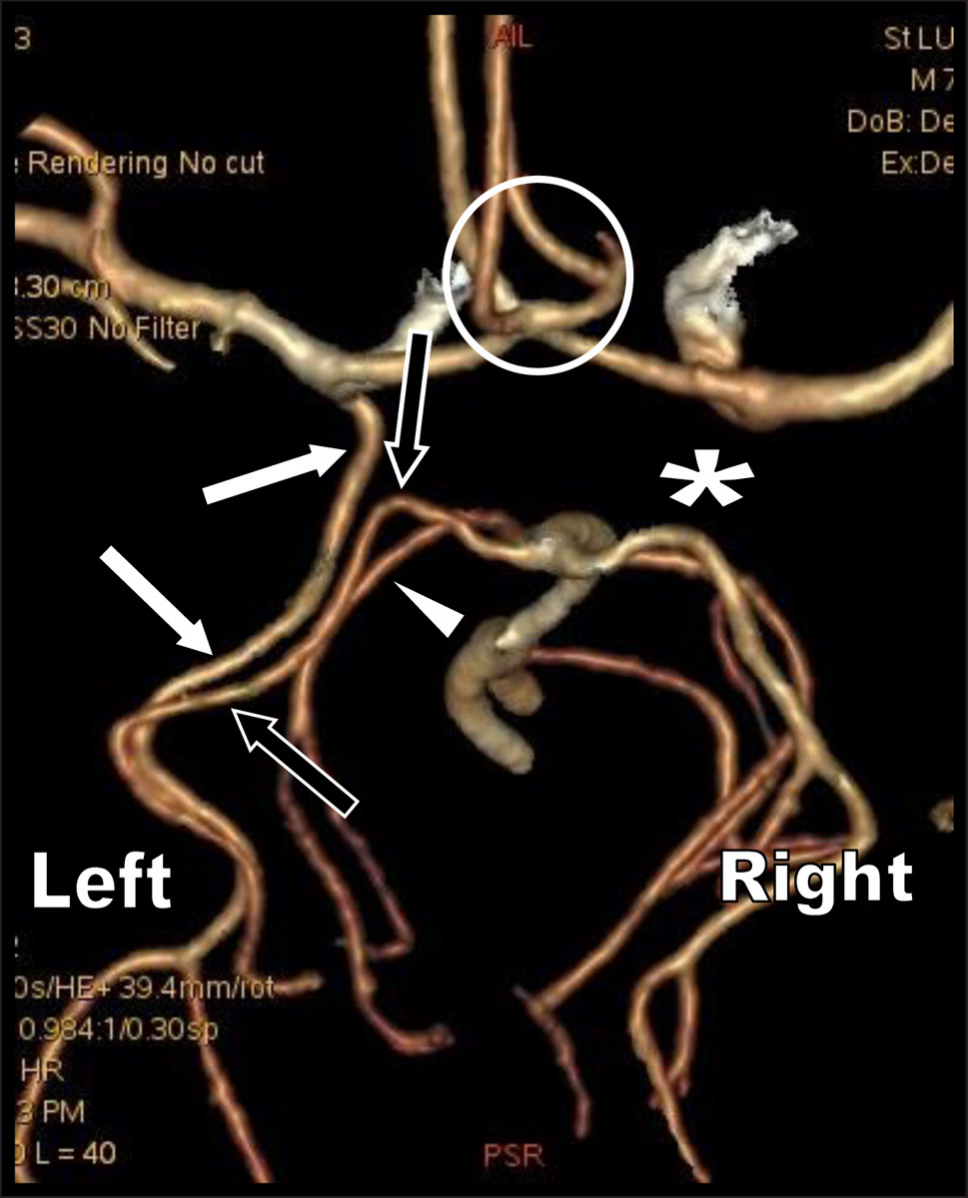
3D volume rendering view of the Circle of Willis obtained from the CT arterial phase. View from the vertex (left side is thus on the right of the figure).

**Figure 2:**
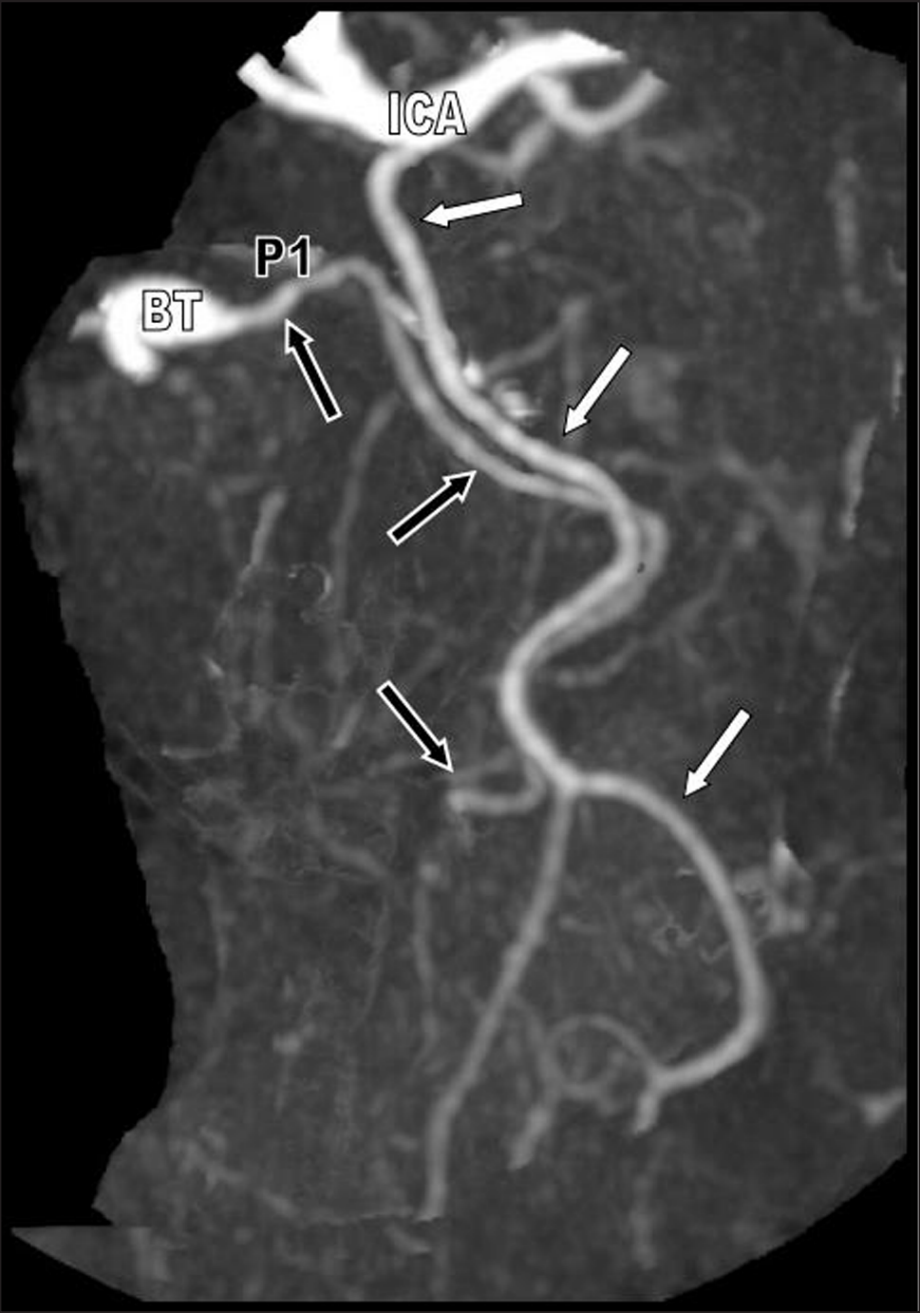
Selective oblique Maximal Intensity Projection (MIP) reformation of the duplicated left posterior cerebral arteries.

A 78-year-old patient with a recent history of transitory ischemic attack with right upper extremity weakness was referred for computed tomographic angiography (CTA) of the neck arteries. No significant stenosis was found, but systematic 3D study of the circle of Willis (Figure 1) revealed a very unusual variant pattern of the left posterior cerebral artery (PCA) which appeared duplicated. The classical and well-differentiated P1 segment of the left PCA (black arrows) emerging from the basilar trunk (BT) appeared, coupled with another large artery (white arrows) emerging from the internal carotid artery and having a parallel path to the occipital lobe. This situation was distinct from a classical large posterior communicating artery (PcoA). The superior cerebellar artery was also well demarcated (small white arrowheads). There was no right PcoA (white asterisk) and trifurcation of the anterior cerebral artery was also found (white circle). The PCA was supplying the calcarine artery and the atypical aberrant parallel artery was supplying the parieto-occipital area (Figure 2). This variant was recognized as a large persistent anterior choroidal artery realizing and exceedingly rare variant of the PCA called a true “fetal PCA”.

## Comment

The vocable of “fetal posterior cerebral artery” (FPCA) encompasses a group of developmental variants of the posterior cerebral artery (PCA) in which a significant portion of the distal PCA remains perfused through a branch of the internal carotid artery (ICA).

When a FPCA variant is present, thromboembolism in the ICA may result in paradoxical PCA territory infarction (with or without concomitant infarction in the classical ICA territory) [1].

During the choroidal stage of embryogenesis (at five weeks of gestation) the anterior brain circulation is constituted by two main branches of the ICA: the rostral branch (rICA) and the caudal branch (cICA) which prefigures the future posterior communicating artery (PcoA).

The rICA feeds the anterior carotid artery (ACA) and the anterior choroidal artery (AchoA) and the cICA feeds the posterior choroidal artery (PchoA) and the future P1 segment of the PCA.

During growth of the posterior cerebrum, cerebellum and brainstem acceleration arises in the development of the posterior arterial network. It progressively becomes independent from the anterior circulation and the primitive transitory carotid-basilar anastomoses completely regress.

**Figure 3:**
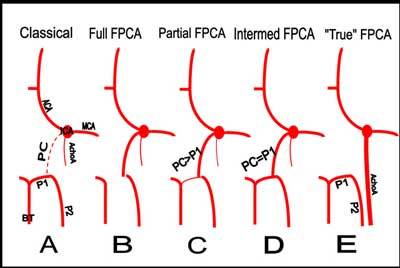
Schematic view of the different variants of PCA. PC = PcoA.

Different scenarios explain the pattern of the future definitive PCA. Most commonly (Figure 3A) the middle cerebral artery (MCA) and the PCA predominantly develop and take over the previously dominant choroidal arteries (they regress but usually do not completely disappear). The caudal branch of the ICA also regresses becoming the PcoA (that may also completely disappear).

Another scenario produces the common FPCA variants (Figure 3B, C and D). The caudal branch of the ICA doesn’t regress but clearly remains dominant as a large PcoA (it becomes the proximal segment of the FPCA variant). Depending of the caliber of the P1 segment of the PCA we may distinguish a “full PPCA variant” (Figure 3B) (P1 is nonexistent or not visible), a “partial FPCA variant” (Figure 3C) (P1 is smaller than the PcoA) or an “intermediate FPCA variant” (Figure 3D) (P1 and the PcoA have the same calibre).

A third but extremely rare scenario called the “true fetal PCA” variant (Figure 3E) is reported in our patient. Two independent PCAs will emerge. One PCA (often dominant) derives from a persistent large primitive AchoA and the second PCA (often smaller) develops in the usual way.

## References

[1] Lambert, SL, Williams, FJ, Oganisyan, ZZ, Branch, LA and Mader, EC, Jr. Fetal-Type Variants of the Posterior Cerebral Artery and Concurrent Infarction in the Major Arterial Territories of the Cerebral Hemisphere. J Investig Med High Impact Case Rep. 2016 9 13; 4(3). eCollection 2016 Jul–Sep. DOI: 10.1177/2324709616665409 PMC5024744 27660767

